# Clinical Lecture on Infantile Paralysis, Etc.

**Published:** 1872-04

**Authors:** Richard Barwell

**Affiliations:** Surgeon to Charing-Cross Hospital


*Clinical Lecture on Infantile Paralysis and its Resulting Deformities.* By RICHARD BARWELL, F.R.C.S., Surgeon to Charing-Cross Hospital.

GENTLEMEN: Before showing you one or two cases and reading to you certain short extracts from my note-book, it seems desirable that I should define the class of malady which I intend to bring before you. Infantile, like adult, life is liable to certain diseases of the brain and spinal cord which result in loss of power in one or more limbs. But, besides these, the infant is subject to a form of paralysis unattended by any symptom of illness—certainly of severe illness; and this, the class of case I propose for your study, being peculiar to the infant, alone deserves the name of "infantile paralysis." Other paralytic maladies, whether occurring in senility, in the prime, or in infancy, should be classed in the categories of meningitic, myelitic, lead-poisoning, traumatic, or other form of disease. But to enable us to separate one thing from a heap of others, we must have means of recognizing that single object whenever we may see it. Hence there devolves on me the ungrateful task of making a definition out of mere negatives; for the typical, and, in my experience, the common, form of the disease is, that the child should be put to bed perfectly well at night, and next morning be taken up with loss of power over a limb or part of a limb, yet otherwise quite well or only very slightly ailing.

For example: This child, Eva H., aged 13 (March, 1871), looks extremely healthy, except that she is lame from paralysis of the left leg. Her mother, an intelligent, clear-headed woman, says: "When she was about eighteen months old I took her out of bed one morning and put her on the floor to walk. She tumbled down. I thought she was stupid, and put her on her feet again. She fell at once, and I then found her leg was paralyzed. She was not at all ill or fretful, either the night before or that morning; but I think that side of her body was perhaps a little tender."

Agnes P., whom you have often seen, was, when she first came, nine months old, a plump and as healthy a baby as ever I saw, except loss of power in the deltoid of the right arm. The mother says: "Two months ago I took the baby out of bed, and it did not move the arm. I showed it to the doctor, and he said the child must have been hurt. I do believe the girl I had then lifted it up by the arm; but the baby did not cry either the night before or that morning more than usual. She always cried when she was being undressed and washed. I observed no difference in that, and she seemed quite well."

One more history from my note-book:

Louisa R., brought to me in April, 1870, when seven years and a half old, with foot deformity resulting from paralysis. Her mother, an active-minded and very acute woman, says that one very hot day, when the child was about eight months old, she was put asleep under a tree on the lawn, and on a damp cushion. The next day she was taken up with the right leg paralyzed. The child had not then any sign of illness whatever, and always has been, with that exception, a strong, healthy girl.

Of such cases and histories I could give you almost an unlimited number. Some attacks may have been preceded for an hour or two, perhaps even for a day or two, by some slight disturbance, fretfulness, a hot skin or mouth, even a slight convulsion. On the morning of the discovery there may be some evidence of pain in the affected limb, some feverish disturbance, and the disease itself may affect a larger or smaller portion of the body. But the more the history and concomitants depart from the type which I have given above, the more any disturbance approaches absolute illness —especially if convulsions have been frequent and severe—the more the stricken regions resemble the relationship of hemiplegic or paraplegic parts, the more likely is the case to be an example of myelitis or meningitis, and the less likely to belong to the pure infantile paralysis, which, as you have seen, does not exhibit a single symptom of central nervous disease.

I feel bound to insist strongly on this distinction, because medical opinion has of late years more and more agglomerated around the idea, fallacious in origin and in result disastrous, that all these cases are dependent on cerebral or spinal disease. Heine, Fliess, Laborde, Radcliffe, and many others, have upheld this notion; and I must shortly point out what essential differences separate this disease from spinal affections. But first, as to pathological appearances. Only seven cases of anatomical investigations have been published. In four of these nothing was detected; in one, that of Dr. Fliess (*Journal fur Kinderkrankheiten*), was found a congestion which, emanating from the gums of the teething child, was continued on to the membranes of the cord. It need hardly be pointed out that the preconceived ideas which aided this description are more easily comprehensible than the anatomy of the congestion. To the other two post-mortem examinations I must unfortunately also take exception, for the two cases which form the groundwork of Dr. Laborde's treatise, "La Paralysie (dite Essentielle) de l'Enfance," are cases respectively of myelitis and meningo-myelitis, with their characteristic histories and symptoms, and do not belong to the disease under consideration. The absence of illness and of danger to life renders it improbable that morbid anatomy will ever furnish a clue to the causality of this disease; we must, at all events for the present, be content with studies from life.

The children we have now to do with are not ill, have no severe convulsion, insensibility, nor pain; therefore no disease marked by severe symptoms, such as myelitis or meningitis, can be present. But a congestion of the cord does not give rise to any attack of such suddenness and severity; it may also be supposed to vanish at death after having produced paralysis in life. There is, too, a certain similitude between congestive and infantile paralysis—such as immunity of the respiratory muscles, bladder, and rectum. Nevertheless, when the condition of a patient with congestion of the spinal cord is compared with a case of infantile paralysis, the observer will not fail to mark a vast difference between the plump limber limbs of the one, and the cold, shriveled, flaccid muscles of the other.

A further investigation into the condition, more especially by various modes of electricity, will render prominent other and more radical differences. A rapid view may best be given by arranging these into opposite columns.

| Spinal Congestion.* | Infantile Paralysis. |
| --- | --- |
| Paralysis incomplete—always at first, and generally throughout the case ; it tends, however, to deepen. | Nothing can be more utter and entire than the loss of power at the very commencement ; it tends, however, to lighten. |
| Generally paraplegic in form at the end, if not at the beginning. Usually one leg is attacked first, then the other. The malady becomes more extensive as it goes on. | Paraplegic form is not usual at the commencement, still less afterwards. When the disease has attacked one part, it never subsequently affects another. It is most extensive at its commencement. |
| Tingling in fingers and toes. | I have never been able to detect the slightest sign of pain or discomfort. In a few cases there is a little and a doubtful increase of sensibility to touch. |
| Shortness of breath not infrequent. | No alteration ever observed in breathing. |
| Fluctuations towards better and worse occur in all cases ; sometimes these are rather frequent. | No change, backward or forward, ever occurs ; if the patient gain a step, he does not lose it again. (Effects produced by treatment are not referred to.) |
| There is a decided tendency to relapse, even after the patient appears to have got well. | No tendency to relapse. I have never seen or heard of a limb being paralyzed a second time, nor of another limb being the subject of a second attack. |
| Reflex irritability is, according to some authorities, increased—according to others, decreased. | Reflex irritability is so utterly annihilated as to leave no room for variety of opinion. |
| No wasting of paralyzed muscles. | Extremely rapid wasting of paralyzed muscles, setting in very quickly after the attack. |
| No marked impairment in affected muscle of electro-contractility. | Remarkable impairment of electro-contractility—indeed, total loss of reponse to the induced current. |

These differences are very marked, and so important that the first six alone appear to separate nosologically the one disease from the other; but it is to the last three that I would especially direct attention. They are physiologically connected in infantile paralysis. The induced current ceases entirely to call forth any contractions of the paralyzed muscles in from twelve to seventy-two hours. At the same time there is developed an abnormal sensibility to the voltaic current. Now this peculiar behavior never occurs in cases of central paralysis, but is exactly what happens when a nerve supplying a muscle has been divided; moreover, both muscles—i. e., the one whose nerve has been cut and the one paralyzed by this malady—lose all excito-motor contractility. But when a nerve of a warm-blooded animal is cut,

* The symptoms of spinal paralysis are taken from Reynolds's System of Medicine, vol. ii. p. 663 *et seq.*

the muscle, at first flaccid, becomes very hard and strongly contracted as long as the wound remains unhealed. When this is well, the muscle relaxes. The nearer to the spine be the wound the harder and more contracted will be the muscle, and the less rapid is the loss of sensibility to the induced current. If we divide the nerve close to its entrance into the belly of the muscle we find hardly any rigidity, quick loss of galvano-contractility, and rapid wasting. Therefore, if we could go a little further and destroy the nerve absolutely at its distribution among the muscular fibres, we should, in all probability, carry the above three characteristics of infantile paralysis—namely, flaccidity, irresponse to faradaism, and wasting—as far as they are developed by the disease itself.

It appears impossible to escape the conclusion that infantile paralysis is a malady purely peripheral, affecting the ultimate nervous fibrillæ of distribution in the muscular elements. I am anxious, gentlemen, that this conclusion be not pushed too far. I am not supposing or imagining any degenerative or structural change in the nerve-fibres as the cause of this malady. The attack is too rapid and too quickly succeeds exposures to warrant such an idea. The essence of the disease probably lies in some subtle derangement of relationship between ultimate nerve and ultimate muscle-fibre: perhaps from some inflammatory, but more probably from a chemical or nutrient change. In after years any amount of tissue degeneration may occur.

Allow me now to refer to a former remark, and to explain its intention. A few paragraphs ago I said that the prevalent notion concerning the central origin of this malady was in its results disastrous, and in saying this I refer especially to the effects produced by loss of time. In the first few hours of the attack the affected muscles are still sensitive to the faradaic or interrupted current. This sensibility rapidly declines, and I am not aware that any use of this force would prevent its extinction. After a time, which varies in different cases, there is established a sensibility to the voltaic or battery current, and it is upon this that we must rely as our chief aid in the treatment of the disease; and a judicious practice, by keeping alive this contractility, preserves the muscle from extreme wasting and degeneration. To wait, with temporizing treatment, or with none at all, until this sensibility is, in its turn, extinct, is the disastrous result of the belief I have combated. Delay in applying the treatment pays a heavy percentage in subsequent difficulty and duration. Very few cases get well of themselves, and tissue change, as evidenced by the wasting, begins almost immediately.

If then, a child, within twenty-four hours of its first attack, do not already show marked signs of recovery, not merely in one or two, but in every muscle, the state of the case should be carefully and rigorously tested by faradization, and again in another twentyfour or forty-eight hours. Any muscle which, at the end of thirty-six or seventy hours, has ceased to respond, or responds more feebly than at the last examination, will not of itself get well, and to delay treatment is in all probability to condemn the patient to a crippled existence. No disease is so obstinate when old; but while still new, few are more amenable to treatment.—*London Lancet.*

